# Variation on a Theme: Vibrational Signaling in Caterpillars of the Rose Hook-Tip Moth, *Oreto rosea*

**DOI:** 10.1673/031.010.5401

**Published:** 2010-06-04

**Authors:** Jaclyn L. Scott, Sarah M. Matheson, Jayne E. Yack

**Affiliations:** Department of Biology, Carleton University, Ottawa, ON KIS 5B6, Canada

**Keywords:** acoustic, caterpillar, communication, territoriality, evolution, spacing

## Abstract

Abstract Vibrational communication in hook-tip moth caterpillars is thought to be widely used and highly variable across species, but this phenomenon has been experimentally examined in only two species to date. The purpose of this study is to characterize and describe the function of vibrational signaling in a species, *Oreta rosea* Walker 1855 (Lepidoptera: Drepanidae), that differs morphologically from previously studied species. Caterpillars of this species produce three distinct types of vibrational signals during territorial encounters with conspecifics — mandible drumming, mandible scraping and lateral tremulation. Signals were recorded using a laser-doppler vibrometer and characterized based on temporal and spectral components. Behavioural encounters between a leaf resident and a conspecific intruder were staged to test the hypothesis that signaling functions as a territorial display. Drumming and scraping signals both involve the use of the mandibles, being hit vertically on, or scraped laterally across, the leaf surface. Lateral tremulation involves quick, short, successive lateral movements of the anterior body region that vibrates the entire leaf. Encounters result in residents signaling, with the highest rates observed when intruders make contact with the resident. Residents signal significantly more than intruders and most conflicts are resolved within 10 minutes, with residents winning 91% of trials. The results support the hypothesis that vibrational signals function to advertise leaf occupancy. Signaling is compared between species, and evolutionary origins of vibrational communication in caterpillars are discussed.

## Introduction

Acoustic communication in adult Lepidoptera has been broadly studied and serves a variety of social and defensive functions (Minet & Surlykke 2003). However, research on acoustic communication in larval Lepidoptera is currently limited. Caterpillars rely on communication during various stages of their life cycles for foraging, defense, aggregation, shelter building, or resource competition (Costa & Pierce 1997; Fitzgerald & Costa 1999; Cocroft 2001; Costa 2006), but little is known about the mechanisms used to broadcast and receive signals (Costa & Pierce 1997). Vision seems unlikely to be an important sensory modality because caterpillars have simple eyes, capable of discriminating only crude images (Warrant et al. 2003). Consequently, most studied caterpillar communication systems focus on chemical and tactile modalities, where such cues are used mainly in species traveling in processions (Fitzgerald 1995; Ruf et al. 2001; Fitzgerald & Pescador-Rubio 2002).

There is increasing evidence that larval Lepidoptera employ an acoustic sense for communication, primarily in the form of vibration. Although anecdotal reports (e.g. Federley 1905; Dumortier 1963; Hunter 1987) suggest that the phenomenon is widespread, experimental evidence for vibrational communication in caterpillars is limited. Lycaenidae and Riodinae butterfly larvae use vibrations to maintain mutualistic relationships with ants (DeVries 1991; Travassos & Pierce 2000). Vibrations are also employed in territorial encounters with conspecifics in four species of moth larvae (*Sparganothis pilleriana,* Russ 1969; *Drepana arcuata,* Yack et al. 2001; *Caloptilia serotinella,* Fletcher et al. 2006; and *Drepana* *bilineata,* Bowen et al. 2008). Further research characterizing and testing the function of vibrational signaling in caterpillars is necessary for understanding its ubiquity and role in different families of Lepidoptera.

**Figure 1.**
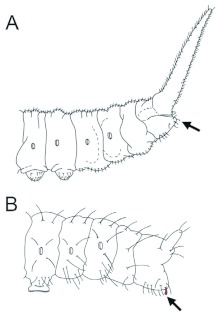
Schematic drawings of the two morphological forms found in the Drepaninae. (A) *Oreta rosea*, representative of the form lacking anal oars. A pair of setae, PPI (red), is found in the region where anal oars would be located. (B) *Drepana arcuata,* representative of the form that possesses a pair of modified setae referred to as “anal oars” (red). Both forms lack anal prolegs. Modified from Stehr (1987). High quality figures are available online.

Drepaninae, the largest subfamily of moths belonging to the Drepanidae (Minet & Scoble 1999), offers a unique opportunity for studying the function and evolution of vibrational communication in caterpillars. Although vibrational signaling has only formally been described in two species to date (*D. arcuata* and *D. bilineata*), there is abundant suggestive evidence (Dyar 1894; Federley 1905; Nakajima 1970, 1972; Bryner 1999; Sen & Lin 2002; I. Hassenfuss, personal communication) that it is common and highly variable in the Drepaninae. Variation exists in the signal-producing structures, types of signals produced and territorial behaviour. Both species experimentally studied to date employ vibrational communication to resolve territorial disputes with conspecifics over silk leaf shelters (Yack et al. 2001) or leaf territories (Bowen et al. 2008). Both possess specialized sound-producing structures, a pair of modified setae (anal oars) on their terminal abdominal segment, to produce vibrational signals. There is evidence that many other Drepaninae species possess anal oars, which can be highly variable in both shape and size across species (Fig. 1A; Nakajima 1970, 1972). Other species lack anal oars altogether (Fig. 1B) and may completely lack vibrational signals. Signaling in this second morphological form has yet to be experimentally analyzed.

The goal of this study is to examine one of these species, *Oreta rosea,* a sympatric congener of *D. arcuata* and *D. bilineata* that lacks anal oars (Fig. 1A). To the authors' knowledge, there are no reports to date on territorial behaviour or vibrational signal production in this species. Since larvae of *O. rosea* live solitarily as late-instars (see Results), we hypothesize that they will exhibit territorial behaviours. If they are territorial, then: (*i*) residents should maintain exclusive use of their territory, (*ii*) residents should defend their territories against conspecifics, and (*iii*) intruders should only rarely displace residents. The aim of this study is to test for territorial behaviour and vibrational signaling, and if present, compare it with previously studied species. Life-history traits relevant to territoriality and spacing will also be compared to provide insight into some of the factors underlying the evolution of signaling in the Drepaninae.

## Materials and Methods

### Animals

*Oreta rosea* Walker 1855 (Lepidoptera: Drepanidae) moths were collected from the wild at ultraviolet collecting lights between May and August 2007 in Dunrobin, Ontario, Canada. Females oviposited on cuttings of viburnum (*Viburnum lentago*) and larvae were reared indoors on *V. lentago* or *V. opulus* under a LD 18:6 photoperiod at 21–26°C. Early- (first and second) and late- (third to fifth) instar larvae were used for life-history and behavioural observations. Late-instars were further used for morphological analysis of sound-producing structures, laser vibrometry recordings and behavioural trials.

### General behaviour and life-history

Behavioural observations relevant to communication and spacing were recorded daily. These included the position on the leaf, presence of silk on the leaf, mode of feeding, and interactions between individuals. Photographs of eggs, larvae and adults were obtained with an Olympus dissection microscope (SZX12; www.olympus.com) equipped with a Zeiss camera (AxioCam MRc5; www.zeiss.com), or with a Nikon Digital SLR camera (D80; www.nikon.com).

### Signal characteristics

Vibrational signals were monitored and characterized using two recording methods - a microphone and laser-doppler vibrometer (LDV). Both methods involved recording late-instar larvae with a videocamera and a microphone or LDV during encounters with conspecific intruders (see below). Vibrations measured using a Polytec LDV (PDV 100; www.polytec.com) were digitized and recorded onto a Marantz Professional portable solid state recorder (PMD 671; www.marantzpro.com; 44.1 kHz sampling rate). Vibrations perpendicular to the leaf surface were measured at the location of a circular piece of reflective tape (2.0 mm in diameter) positioned 1 – 2.5 cm from the resident caterpillar. All recordings were made in an acoustic chamber (Eckel Industries, www.eckelacoustic.com). These recordings were used to determine the types of signals produced and to measure temporal and spectral characteristics of signaling. Temporal characteristics, including mean signaling bout duration, mean interval duration between signaling bouts and number of signals per bout were measured using Raven Bioacoustics Research Program (Cornell Laboratory of Ornithology; www.birds.cornell.edu/brp/). Bouts were defined as any combination of signals that was preceded and followed by feeding, walking or at least 1 s of inactivity. Durations of each signal type were calculated from 20 individuals. Power spectra were made using a 512-point Fourier transform (DFT, Hann window) in Raven Bioacoustics Research Program. Signals were not filtered and a power spectrum of background noise was included for comparison.

**Figure 2.**
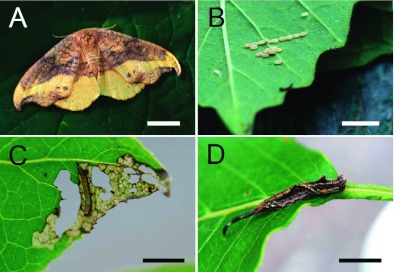
Oreta rosea at various life stages. (A) A female adult moth in resting position, showing the hook-tip wings, characteristic of the Drepaninae subfamily (scale bar = 5 mm). (B) Eggs laid on the underside of a *Viburnum lentago* leaf (scale bar = 5 mm). (C) An early-instar larva that has skeletonized a *V. lentago* leaf (scale bar = 10 mm). (D) A solitary late-instar larva demonstrating the unique physical appearance of *O. rosea* with an elongated caudal projection (scale bar = 10 mm). High quality figures are available online.

### Morphology

Structures associated with signal production and the last abdominal segments (A8–A10) were examined in early- and late-instars preserved in 80% ethanol. For scanning electron micrographs, mandibles and head capsules were dissected, mounted on aluminum stubs and air-dried. Specimens were sputter-coated with gold-palladium and examined using a JEOL scanning electron microscope (JSM-6400; www.jeol.com).

### Signal function

Once it was established that *O. rosea* produces vibrational signals, we tested the hypothesis that signaling functions to advertise occupancy of leaves. Twenty-two encounters were staged between a resident larva and an introduced conspecific intruder of approximately the same size, as described in Bowen et al. (2008). Briefly, late-instar larvae were selected at random from 2 broods of wild-caught females. Residents and intruders were isolated on a leaf or in a container with viburnum twigs, respectively, for at least 30 min prior to the trial. Leaves were chosen based on size (mean ± SD: 8.4 ± 2.1 × 3.4 ± 1.2 cm) and the absence of feeding scars, or other types of leaf damage. Trials were videotaped from 1 minute before the intruder was introduced until 1 min after one contestant left the leaf (i.e. when one contestant ‘won’ the encounter). If there was no winner within 30 minutes, the trial was deemed a ‘tie’. This time was chosen based on previous trials with related species (*D. arcuata,* Yack et al. 2001; *D. bilineata,* Bowen et al. 2008). After each trial, the weight of each caterpillar was recorded and individuals were isolated in a separate container so they would not be reused in another trial. All trials were recorded using a Sony High Definition Handicam (HDR-HC7; www.sony.com) and a remote Sony audio microphone (ECM-MS907) placed 1–2 cm behind the leaf or with the LDV.

Videotapes from 22 trials were analyzed to measure the durations and outcomes of contests, and to monitor changes in signaling rates in both residents and intruders throughout each trial. Durations of trials in which the intruder signaled were compared to those in which only the resident signaled using a Wilcoxon rank sum test. To compare average signaling rates of residents and intruders during encounters, signals from 21 encounters (excluding one trial where the intruder won) were counted at 5-s intervals during the 80-s period prior to and the 80-s period following the time at closest distance between the resident and intruder. The distances between the head of the intruder and closest point of the resident were measured at each interval using ImageJ software (http://rsb.info.nih.gov/ij/).

In 18 trials where the intruder came within at least 0.5 cm of the resident, signaling rates with respect to decreasing distance between individuals were recorded. Rates were measured at three stages — far (20-s interval immediately following the point when the head of the intruder passed the junction of the petiole and the leaf), mid (20-s period following the mid-way point between the far and close distances) and close (20-s period following the point when the intruder first made contact with the resident, or in trials where contact was not made, when the intruder came the closest within 0.5 cm of the resident). Time intervals did not overlap in any of the trials. Signal escalation was analyzed by calculating the mean number of signals at each distance category for each type of signal and each individual. The data were square-root transformed and the means were compared using an analysis of variance (ANOVA). *Post hoc* analyses were conducted using a Tukey-Kramer HSD. A grand mean of signaling rates per signaling type at each distance category was calculated to create a histogram. Overall signaling rates for *O. rosea* were calculated by taking the mean of all signaling types at all distance categories for comparison with *D. arcuata* and *D. bilineata.*

### Comparison with *D. arcuata* and *D. bilineata*

In order to compare signaling between species that possess anal oars and *O. rosea,* signaling rates for *D. arcuata* and *D. bilineata* were obtained from staged encounters from previous studies using similar methods (Yack et al. 2001; Bowen et al. 2008). Types of signals produced, patterns of signaling, signal escalation and signaling rates were compared between species. Overall signaling rates were compared between species using an ANOVA. *Post hoc* analyses were conducted using a Tukey-Kramer HSD.

## Results

### General behaviour and life-history

Adult females (Fig. 2A) lay eggs singly or in small rows of 2–10 on the upper and under surface of the leaf (Fig. 2B). All instars live solitarily on the leaf. Early-instars occupy individual feeding areas at leaf edges, skeletonizing the leaf surface (Fig. 2C). Lateinstar caterpillars occupy their own leaf (Fig. 2D) and will lay down a mat of silk on the leaf surface, but make no shelter. They begin feeding at the tip and will consume almost the entire leaf. If approached by a conspecific, leaf occupants of all instars will produce vibrational signals.

### Signal characteristics

**Figure 3.**
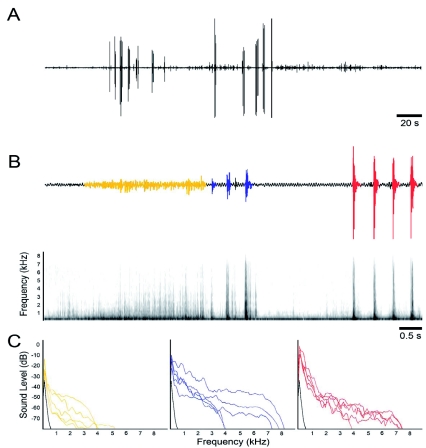
Typical vibrational signals produced during territoriality displays in *Oreta rosea* larvae. (A) Oscillogram of a behavioural encounter illustrating a series of bouts produced by a resident caterpillar when approached by a conspecific intruder from the time the intruder enters the leaf to when it leaves. (B) Oscillogram and corresponding spectrogram of three vibratory signals from one individual - lateral tremulation events (yellow), mandible scrapes (blue), and mandible drums (red). (C) Power spectra of the three vibratory signals (coloured as in part B) from 5 individuals. Background noise is represented in black. High quality figures are available online.

Microphone and LDV recordings revealed that *O. rosea* larvae produce three types of vibrational signals: mandible drumming, mandible scraping and lateral tremulation (Video). Signaling was initiated when a resident of a leaf is approached by a conspecific. Signaling was not ever observed in response to agitating the leaf or disturbances caused by a paintbrush. Overall, signaling typically occurred in bouts (Fig. 3A), lasting 2.2 ± 1.7 s (range = 0.4 – 6.5 s, *n* = 71 bouts from 16 individuals). Bouts typically comprised more than one signal, averaging 4.0 ± 2.0 signals per bout (range = 1.0 — 11.0, *n* = 71 bouts from 16 individuals). Time intervals between bouts were highly variable, ranging from 1.7 – 15.4 s (mean ± SD = 5.1 ± 3.6 s, *n* = 63 intervals from 15 individuals). Spectral analysis revealed that all signals are broadband with most energy ranging from 0.5 – 2.0 kHz (Fig. 3C).

**Video.**
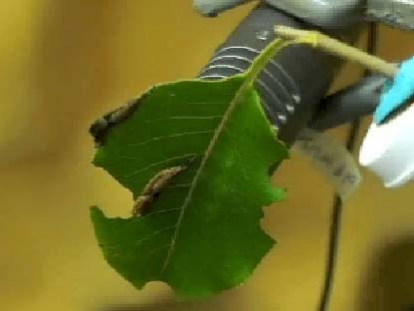
Video clip demonstrating typical vibrational signaling in *Oreta rosea.* High quality figures and the video clip are available online.

**Figure 4.**
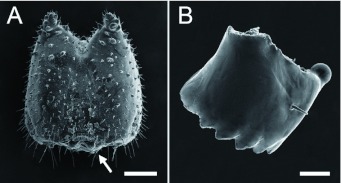
Scanning electron micrographs of sound-producing structures in *Oreta rosea.* (A) An anterior view of a lateinstar larval head capsule showing the position of the left mandible (arrow, scale bar = 500 µm). (B) Higher magnification of a single mandible showing the serrated edge that is scraped against the leaf surface (scale bar = 100 µm), High quality figures are available online.

***Mandible drumming*.** Mandible drumming (Fig. 3) is produced by rapidly hitting the leaf surface with the serrated edges of open mandibles (Fig. 4) to produce a short, percussive signal. Mandible drumming was found to be used more frequently as the intruder approached the resident. The mean ± SD duration of a single drum is 66.9 ± 20.1 ms (range = 41.6 – 119.8 ms, *n* = 71 signals from 19 individuals).

***Mandible scraping*.** Mandible scraping (Fig. 3) is produced by a movement of the head, thorax and first two abdominal segments in a lateral arc in one direction, dragging the mandibles across the leaf surface to produce a scratching noise. Often the caterpillar will scrape in the other lateral direction immediately after the first scrape. Distance and duration of the scrape can be highly variable depending on proximity of the conspecific and other factors, such as proximity of the leaf edge. Mandible scraping was also found to be used more frequently as the intruder approached the resident. The mean ± SD duration of a single scrape is longer than that of a mandible drum, lasting 125.6 ± 21.4 ms (range = 70.0 – 157.2 ms, *n* = 69 signals from 17 individuals).

***Lateral tremulation*.** Lateral tremulation (Fig. 3) was only observed in about half the individuals (in 40.9% of trials) and consists of quick, short, successive lateral movements of the head and thorax while the rest of the body remains motionless. A lateral tremulation event is distinguished from a mandible scrape by its much shorter, highly repetitive movement, where the mandibles never touch the leaf surface. A single lateral tremulation event lasts on average 2.0 ± 0.6 s (range = 1.3 -–3.1 s, *n* = 32 signals from 9 individuals), and although highly variable, is much longer than a single mandible scrape or drum. One lateral tremulation event typically occurred at the beginning of a bout, followed by any combination of mandible drums and scrapes. Bouts rarely contained more than one lateral tremulation event.

**Figure 5.**
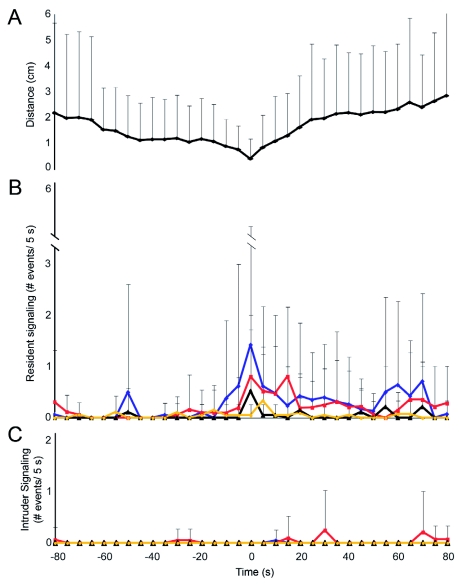
Signaling of resident and intruder larvae 80-s prior to, and following the closest point of contact (measured from the head of the intruder to the closest point of the resident) over 21 encounters. (A) Mean distance (+SD) between resident and intruder larvae at the beginning of each 5-s interval. Signaling rates of residents (B) and intruders (C). Diamonds (blue) denote average mandible scrape rate per 5-s interval, squares (red) denote average mandible drum rate per 5-s interval, circles (yellow) denote average lateral tremulation events per 5-s interval, and triangles (black) denote average lateral tail contact rate per 5-s interval. High quality figures are available online.

**Figure 6.**
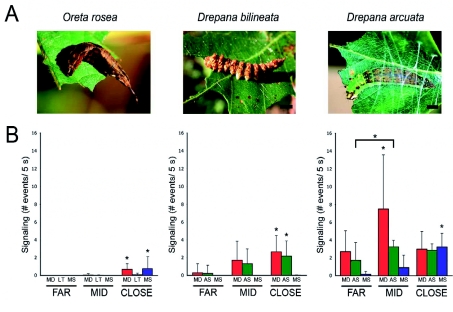
Comparison of signaling between con-familial larvae of *Oreta rosea, Drepana bilineata* and *Drepana arcuata.* (A) Photographs of 0. rosea (far left, scale bar = 3.5 mm), *D. bilineata* (middle, scale bar = 4 mm), and *D. arcuata* (far right, scale bar = 4 mm) late-instar larvae demonstrating differences in appearance and territory investments. (B) Mean (+SD) signal rates of residents with direct contact (or distance between resident and intruder of less than 0.5 cm in *D. bilineata* and *O.* *rosea*) at three stages of intruder approach (*O. rosea*, far left, *n* = 18; *D. bilineata,* middle, *n* = 21; *D. arcuata,* far right, *n* = 16). Asterisks denote significant differences within each signal type, within each species. Overall, signaling rates between all species differ significantly, where *D. arcuata* signals the most, and *O. rosea* the least (ANOVA, *F* = 75.9, *P* < 0.001). Modified from Bowen et al. (2008).High quality figures are available online.

### Signal function

A total of 22 encounters were staged between a resident and an intruder of equal weight. Weights of the contestants ranged from 7.5 – 244 mg (mean = 88.2 ± 74.1 mg, *n* = 44), but were similar between contestants in a given trial (mean difference = 18.0 ± 17.6 mg, paired *t*-test, *t* = 1.23, *P* = 0.23). Residents won 91.0% of trials, intruders won 4.5% and 4.5% were ties. Contests lasted 457.4 ± 330.7 s in trials where a winner was decided (*n* = 21). The only contest won by an intruder was of average duration (510.0 s).

Residents remained silent until they detected an intruder (Figs. 5, 6). Residents signaled in 84.2%) of trials where signaling occurred, and were the first to signal in 78.9% of trials, at a latency of 200.9 ± 193.3 s (*n* = 15) from the beginning of the trial and at a mean distance of 6.97 ± 9.91 mm (*n* = 15) from the intruder's head to the closest point on the resident's body. Residents remained in the same approximate position on the leaf during trials. Signaling did not occur at all in three trials. Intruders signaled in 47.4% of trials where signaling occurred, but were the only contestants to signal in 15.8% of trials. Overall, residents signaled at significantly higher rates than intruders (Fig. 5; paired *t*test, *t* = -3.84, *P* = 0.001, *n* = 21).

The rate of signaling in residents escalated as the intruder approached (Fig. 6B). Very little signaling was observed at far and mid distances, except for the occasional mandible drum and lateral tremulation event. Overall, signaling was significantly higher at close distances, where both mandible drumming and mandible scraping did not change from far to mid distances but increased significantly from mid to close distances (Fig. 6B; ANOVA; MD: *F* = 22.6, *P* = 0.001; MS: *F* = 6.1, *P* = 0.43; V: *F* = 22.6, *P* < 0.001). Lateral tremulation did not vary significantly with distance, perhaps due to the fact that it was rarely observed in comparison to the other signals (Fig. 6B; ANOVA, *F* = 2.8, *P* = 0.07).

A fourth type of behaviour that lacks a vibrational signal was observed in 31.8 *%* of trials (Fig. 5). Lateral tail contact involves a quick lateral movement of the elongated caudal projection, usually towards the intruder. Lateral tail contact is typically observed when the intruder touches the resident near its abdominal end, and the resident swings its tail back and forth multiple times, making contact with the intruder. Lateral tail contact was found to increase significantly from mid to close distances (ANOVA, *F* = 4.9, *P* = 0.01). Biting was never observed.

### Comparison with *D. arcuata* and *D. bilineata*

*Drepana arcuata, D. bilineata* and *O. rosea* are all solitary in their late-instars and defend territories against conspecifics. *D. arcuata* is the only species that makes a silken leaf shelter, while the others produce minimal silk by laying mats on the leaf surface (Fig. 6A). Morphological analyses revealed that the mandibles are similar in position and general appearance between species, and confirms the lack of anal oars in *O. rosea,* which are present and important signal producing structures in *D. arcuata* and *D. bilineata.* Consequently, *O. rosea* does not produce an anal scraping signal. It does, however, produce a lateral tremulation signal, which is not found in either of the other species (Fig. 6B). Mandible drumming is produced by all species and mandible scraping is produced in *O. rosea* and *D. arcuata* (Fig. 6B). Signaling patterns are similar between species, all occurring in bouts, although the structure of bouts differs. The pattern of signaling within bouts in *O. rosea* is highly variable, whereas patterns of signaling in *D. bilineata* and *D. arcuata* are more regular, often beginning with an anal scrape followed by one or more mandible drums/scrapes.

In terms of signaling rates, *O. rosea* signals significantly less than *D. arcuata,* producing significantly fewer mandible drums and mandible scrapes (Fig. 6B; ANOVA; MD: *F* = 41.1, *P* < 0.001; MS: *F* = 30.1, *P* < 0.001). When compared to *D. bilineata, O. rosea* mandible drums significantly less (ANOVA, *F* = 41.1, *P* < 0.001). Lateral tail contact was also compared between species, and it was found that *O. rosea* contacts conspecifics with its caudal projection at similar rates to *D. bilineata* (Independent Mest, *t* = -0.61, *P* = 0.54, two-tailed) whose caudal projection is about 10 times smaller (Fig. 6A). Unlike *D. arcuata* and *D. bilineata, O. rosea* was typically not observed to contact a conspecific with its head. Combined signaling rates between species (not including lateral tail contact) differ significantly between species, *D. arcuata* signaling significantly more than *D. bilineata* and *O. rosea,* and *D. bilineata* signaling significantly more than *O. rosea* (ANOVA, *F* = 75.9, *P* < 0.001).

## Discussion

The purpose of this study was to examine a variation on a theme — vibrational signaling in hook-tip moth caterpillars. The Drepaninae subfamily shows interesting diversity in vibrational signaling and morphology of the terminal abdominal segment. While all species lack anal prolegs (Minet & Scoble 1999), only some possess specialized soundproducing structures, anal oars. The present study is the first to describe vibrational signaling in a species of Drepaninae that does not possess these structures.

Despite the lack of anal oars, our results show that *O. rosea* produces three types of substrate-borne signals upon encountering a conspecific — mandible drumming, mandible scraping and lateral tremulation. The only morphological structure employed by *O. rosea* to produce vibrational signals are the mandibles, which do not appear to be specifically differentiated for sound production. There is mounting evidence demonstrating that the use of mandibles for acoustic signaling may be common in caterpillars (Yack et al. 2001; Brown et al. 2007; Fletcher et al. 2006; Bowen et al. 2008; Bura et al. 2009). Although mandible drumming and scraping have already been described in two other species of Drepaninae (Yack et al. 2001; Bowen et al. 2008), lateral tremulation has not been reported until now.

### Signal function

Results from staged encounters support the hypothesis that vibrational signaling in *O. rosea* is used to advertise occupancy of leaf territories. Our findings are also consistent with territorial displays in other animals (Huntingford & Turner 1987). Signaling is produced in the presence of a conspecific and acoustic displays are restricted to a territory. Residents are typically the first to signal during an encounter, signaling significantly more than intruders, and winning significantly more encounters (more than 90% of trials in this study). Signaling rates also escalate as the intruder approaches. Alternative signal functions observed in other acoustically communicating larvae include aposematic warning signals (Brown et al. 2007; Bura et al. 2009), mutualistic relationships with ants (DeVries 1991; Travassos & Pierce 2000) and conspecific recruitment (Fletcher 2007). The aposematic signaling hypothesis can be discounted in *O. rosea* larvae because they have no obvious noxious defenses and are palatable to predators (e.g. predatory stink bugs, leopard geckos, tarantulas; unpublished data). Furthermore, *O. rosea* larvae were not observed to produce vibrational signals during encounters with predators (unpublished data). The latter alternative hypotheses can also be discounted in *O. rosea* larvae as they do not produce secretions, are not associated with ants and are not gregarious at any stage of their life cycles. In the future, playback experiments may provide further insight into the function of vibrational signaling in these caterpillars.

### Comparison between species and insights into evolution

Ritualized vibrational signaling in *O. rosea* and other species of Drepaninae is thought to have evolved to avoid the costs associated with physical fighting, as territorial encounters in other larval species often end in serious injury or death to one of the contestants (Weyh & Maschwitz 1982; Okuda 1989; Berenbaum et al. 1993). The investment in leaf defense may be proportional to investment in nest production, because leaf shelters are expensive to build and valuable to own (Berenbaum et al. 1993; Cappuccino 1993; Costa & Pierce 1997). This is exemplified in the Drepaninae studied to date, where *D. arcuata,* the only species that produces a leaf shelter, invests significantly more in leaf defense via vibrational signaling than *O. rosea* and *D. bilineata.* Of the three species, *D. arcuata* is also the only species that lives gregariously in the early-instar stage. Therefore, the chances of encountering a wandering caterpillar from the same brood is expected to be higher in *D. arcuata* than in *O. rosea* and *D. bilineata* because the latter disperse earlier in development. Therefore, higher rates of ritualized signal production in *D. arcuata* may have evolved to avoid incurring physical injury to relatives.

*Oreta rosea* and *D. bilineata* share similar life-histories in that they live solitarily at all instars, do not build leaf shelters, and produce comparable amounts of silk. Signaling rates would thus be expected to be similar between these two species if signaling was linked to nest investment. However, this is not the case as *D. bilineata* signals at a significantly higher rate than *O. rosea.* To determine the cause for the difference in signaling rates between these two species, future comparative studies examining caterpillar behaviour in natural conditions are required to assess other lifehistory traits that may be linked to signaling. Comparison of territorial behaviour in *O. rosea* and *D. bilineata* also suggests that the elongated caudal projection found in *O. rosea* did not evolve for a defensive function against conspecifics because no significant difference was found in rates of lateral tail contact between species, despite the distinct difference in caudal projection size. This does not discount its use as a defense against heterospecifics, such as parasitoids, and further studies examining its specific function are needed.

The present study contributes to the understanding of vibrational signaling in the Drepaninae, describing signaling in a novel morphological form. It also provides evidence that signaling in the Drepanoidea may be widespread and highly variable. Each species possesses unique characteristics that can contribute to their vibrational signaling repertoire. Further behavioural and morphological observations in a number of Drepanoidea species mapped onto a molecular phylogeny are now underway and will provide additional insights into the ultimate and proximate mechanisms underlying the evolution of ritualized signaling in these caterpillars.

## Abbreviations

MD: mandible drum;
MS: mandible scrape;
LT: lateral tremulation event;
LDV: laser-doppler vibrometer

## References

[bibr01] Berenbaum MR, Green ES, Zangerl AR (1993). Web costs and web defense in the parsnip webworm (Lepidoptera: Oecophoridae).. *Environmental Entomology*.

[bibr02] Bowen JL, Mahony SJ, Mason AC, Yack JE (2008). Vibration-mediated territoriality in the warty birch caterpillar *Drepana bilineata*.. *Physiological Entomology*.

[bibr03] Brown SG, Boettner GH, Yack JE (2007). Clicking caterpillars: Acoustic aposematism in *Antheraea polyphemus* and other Bombycoidea.. *Journal of Experimental Biology*.

[bibr04] Bryner R, Ligue Suisse pour la protection de la nature (1999). Drepanidae — Drépanidés.. *Les Papillons et leur Biotopes: Especes; Dangers qui les menacent; Protection. Suisse et regions limitrophe*..

[bibr05] Bura VL, Fleming AJ, Yack JE (2009). What's the buzz? Ultrasonic and sonic warning signals in caterpillars of the great peacock moth (*Saturnia pyri*).. *Naturwissenschaften*.

[bibr06] Cappuccino N (1993). Mutual use of leafshelters by lepidopteran larvae on paper birch.. *Ecological Entomology*.

[bibr07] Cocroft RB (2001). Vibrational communication and the ecology of group-living, herbivorous insects.. *American Zoologist*.

[bibr08] Costa JT (2006). *The Other Insect Societies.*.

[bibr09] Costa JT, Pierce NE, Choe JC, Crespi BJ (1997). Social Evolution in the Lepidoptera: Ecological Context and Communication in Larval Societies.. *Social Behaviour in Insects and Arachnids*.

[bibr10] DeVries PJ (1991). Call production by myrmecophilous riodinid and lycaenid butterfly caterpillars (Lepidoptera): morphological, acoustical, functional, and evolutionary patterns.. *American Museum Novitates*.

[bibr11] Dumortier B, Busnel RG (1963). Morphology of sound emission apparatus in Arthropoda.. *Acoustic Behaviour of Animals*.

[bibr12] Dyar HG (1894). Classification of Lepidopterous Larvae.. *Annals of the New York Academy of Science*.

[bibr13] Federley H (1905). Sound produced by Lepidopterous larvae.. *Journal of the New York Entomological Society*.

[bibr14] Fitzgerald TD (1995). *The Tent Caterpillars.*.

[bibr15] Fitzgerald TD, Costa JT, Detrain C, Deneubourg JL, Pasteels JM (1999). Collective behavior in social caterpillars.. *Information Processing in Social Insects*.

[bibr16] Fitzgerald TD, Pescador-Rubio A (2002). The role of tactile and chemical stimuli in the formation and maintenance of the processions of the social caterpillar *Hylesia lineata* (Lepidoptera: Saturniidae).. *Journal of Insect Behaviour*.

[bibr17] Fletcher LE (2007). Vibrational signals in a gregarious sawfly larva (*Perga affinis*): group coordination or competitive signalling?. *Behavioural Ecology and Sociobiology*.

[bibr18] Fletcher LE, Yack JE, Fitzgerald TD, Hoy RR (2006). Vibrational communication in the cherry leaf roller caterpillar *Caloptilia serotinella* (Gracillariodea: Gracillariidae).. *Journal of Insect Behaviour*.

[bibr19] Hunter MD (1987). Sound production in larvae of *Diurnea fagella* (Lepidoptera: Oecophoridae).. *Ecological Entomology*.

[bibr20] Huntingford F, Turner A (1987). *Animal Conflicts.*.

[bibr21] Minet J, Scoble MJ, Kristensen NP (1999). The Drepanoid/Geometroid Assemblage.. *Handbook of Zoology: Lepidoptera, Moths and Butterflies Vol 1: Evolution, Systematics, and Biogeography*.

[bibr22] Minet J, Surlykke A, Kristensen NP (2003). Auditory and sound producing organs.. *Handbook of Zoology, vol IV. Arthropoda: Insecta. Lepidoptera, Moths and Butterflies*.

[bibr23] Nakajima H (1970). A contribution to the knowledge of the immature stages of Drepanidae occurring in Japan (Lepidoptera).. *Tinea*.

[bibr24] Nakajima H (1972). Additions to the knowledge of the immature stages of Drepanidae occurring in Japan (Lepidoptera).. *Tinea*.

[bibr25] Okuda T (1989). Aggressive characteristics of diapausing larvae of a stem borer, *Busseola fusca* Fuller (Lepidoptera: Noctuidae) in artificially crowded conditions.. *Applied Entomology and Zoology*.

[bibr26] Ruf C, Costa JT, Fiedler K (2001). Trail-based communication in social caterpillars of *Eriogaster lanestris* (Lepidoptera: Lasiocampidae).. *Journal of Insect Behaviour*.

[bibr27] Russ K (1969). Beiträge zum Territorialverhalten der Raupen des Springwurmwicklers, *Sparganothis pilleriana* Schiff (Lepidoptera: Tortricidae).. *Pflanzenschutzberichte*.

[bibr28] Sen YC, Lin CS (2002). Larval morphology and host plants of Drepanidea (Lepidoptera: Drepanidae) in Southern Taiwan.. *Formosan Entomologist*.

[bibr29] Stehr FW, Stehr FW (1987). Order Lepidoptera.. *Immature Insects*.

[bibr30] Travassos MA, Pierce NE (2000). Acoustics, context and function of vibrational signaling in a lycaenid butterfly-ant mutualism.. *Animal Behaviour*.

[bibr31] Warrant E, Kelber A, Kristensen A, Kristensen NP (2003). Visual Organs.. *Lepidoptera: Moths and Butterflies Vol. 2: Morphology, Physiology and Development*.

[bibr32] Weyh R, Maschwitz U (1982). Individual trail marking by larvae of the scarce swallowtail *Iphiclides podalirius* L. (Lepidoptera; Papilionidae).. *Oecologia*.

[bibr33] Yack JE, Smith ML, Weatherhead PJ (2001). Caterpillar talk: Acoustically mediated territoriality in larval Lepidoptera.. *Proceedings of the National Academy of Science USA*.

